# Guideline adherence in the management of attention deficit hyperactivity disorder in children: An audit of selected medical records in three Australian states

**DOI:** 10.1371/journal.pone.0245916

**Published:** 2021-02-08

**Authors:** Louise A. Ellis, Brette Blakely, Philip Hazell, Sue Woolfenden, Harriet Hiscock, Vanessa Sarkozy, Bronwyn Gould, Peter D. Hibbert, Gaston Arnolda, Hsuen P. Ting, Louise K. Wiles, Charlotte J. Molloy, Kate Churruca, Meagan Warwick, Jeffrey Braithwaite, on behalf of the CareTrack Kids Investigative Team

**Affiliations:** 1 Centre for Healthcare Resilience and Implementation Science, Australian Institute of Health Innovation, Macquarie University, Sydney, New South Wales, Australia; 2 Discipline of Psychiatry, School of Medicine, University of Sydney, Camperdown, New South Wales, Australia; 3 School of Women and Children’s Health, University of New South Wales, Randwick, New South Wales, Australia; 4 Murdoch Children’s Research Institute, Royal Children’s Hospital, Department of Paediatrics, University of Melbourne, Parkville, Victoria, Australia; 5 Tumbatin Developmental Clinic, Sydney Children’s Hospital Network, School of Women and Children’s Health, University of New South Wales, Randwick, New South Wales, Australia; 6 General Practitioner, Paddington, New South Wales, Australia; 7 Australian Centre for Precision Health, Allied Health and Human Performance, University of South Australia, Adelaide, South Australia, Australia; 8 South Australian Health and Medical Research Institute (SAHMRI), Adelaide, South Australia, Australia; Medical University of Vienna, AUSTRIA

## Abstract

**Objective:**

To assess General Practitioner (GP) and pediatrician adherence to clinical practice guidelines (CPGs) for diagnosis, treatment and management of attention deficit hyperactivity disorder (ADHD).

**Method:**

Medical records for 306 children aged ≤15 years from 46 GP clinics and 20 pediatric practices in Australia were reviewed against 34 indicators derived from CPG recommendations. At indicator level, adherence was estimated as the percentage of indicators with ‘Yes’ or ‘No’ responses for adherence, which were scored ‘Yes’. This was done separately for GPs, pediatricians and overall; and weighted to adjust for sampling processes.

**Results:**

Adherence with guidelines was high at 83.6% (95% CI: 77.7–88.5) with pediatricians (90.1%; 95% CI: 73.0–98.1) higher than GPs (68.3%; 95% CI: 46.0–85.8; p = 0.02). Appropriate assessment for children presenting with signs or symptoms of ADHD was undertaken with 95.2% adherence (95% CI: 76.6–99.9), however ongoing reviews for children with ADHD prescribed stimulant medication was markedly lower for both pediatricians (51.1%; 95% CI: 9.6–91.4) and GPs (18.7%; 95% CI: 4.1–45.5).

**Conclusion:**

Adherence to CPGs for ADHD by pediatricians was generally high. Adherence by GPs was lower across most domains; timely recognition of medication side effects is a particular area for improvement.

## Introduction

Attention deficit hyperactivity disorder (ADHD) is the most common mental disorder during childhood, affecting over seven percent of Australian children aged 4 to 17 years [1]. ADHD is a neurodevelopmental disorder characterised by a persistent pattern of inattention, hyperactivity and impulsivity that is pervasive across settings, including home and school [2]. It can be disabling for children in terms of their developmental and social functioning, and some have comorbid behavioural problems, such as oppositional defiant disorder and conduct disorder [1]. The symptoms of ADHD typically persist into early adulthood, and are associated with an increased risk of a range of serious and functionally impairing problems such as difficulties with education, employment, driving, relationships, and criminality [3, 4].

For children with ADHD, early diagnosis and evidence-based treatment improves the chances for positive long-term outcomes [4, 5]. Clinical practice guidelines (CPGs) for ADHD have been developed internationally and become an important framework for evidence-based care. In Australia, the National Health and Medical Research Council (NHMRC) published Clinical Practice Points (CPPs) in 2012 articulating recommended best practice in the diagnosis and management for ADHD.

Despite the availability of CPGs, considerable variation is reported to exist in the diagnostic evaluation and treatment of ADHD [6–9]. This apparent variability has led to concerns among medical and lay communities about overdiagnosis [10–12], especially for boys [13]. Indeed, it has been estimated that in the United States alone, 20% of children diagnosed with ADHD had been misdiagnosed with the disorder [14]. Existing research into health professionals’ ADHD diagnostic and treatment practice has for the most part been undertaken in North America [6, 8, 15–21], with little published research on adherence to CPGs in Europe or Australasia. One of the earliest and most widely known studies [8] of primary care pediatric consultations (n = 401) conducted in 1999 in the United States of America (USA), Canada, and Puerto Rico found that behavioural assessment tools and the Diagnostic and Statistical Manual (DSM) criteria were only used in 37% and 38% of assessments for ADHD, respectively, indicating a lack of standardisation of diagnostic practice. This study focuses on assessment and diagnosis [8] which is broadly reflective of existing ADHD CPG research, with less attention to treatment and ongoing management. Previous studies have also largely used self-reported survey methods [15, 18–20, 22, 23], which are subject to recall and other biases, compared to external medical record review. One of the few chart review studies of 1594 patients across 188 pediatricians in the USA identified poorer adherence to CPGs than reported from studies using pediatrician self-report.

The largest published Australian study examining guideline adherence in the diagnosis of ADHD found self-reported assessment by pediatricians to be largely consistent with best practice, but noted lower rates of comorbidity identification for internalising, externalising and sleep problems in comparison to parent report. However, this study was limited by its reliance on self-report survey data, exclusive focus on diagnostic indicators, participation from a small group of self-selected pediatricians (n = 24) and non-inclusion of general practitioners (GPs), who can also be involved in the care of children with ADHD.

In Australia, unlike many other countries, while GPs are the ‘gate keepers’ to referral, responsible for identifying children with suspected ADHD and referring them to specialist services, they can also play an important role in the ongoing management of ADHD [24]. The importance of ongoing GP involvement in ADHD care has also increased over the years as a result of the rising number of children who present with the disorder [25]. Another recent study examining pediatric practice in Australia undertook audits of 180 Australian pediatric practices in 2008 and 2013, reporting on consultation types and times, comorbid diagnoses, and medications prescribed, but did not capture information about adherence to guidelines among providers treating children with ADHD [26].

Given the serious short- and long-term consequences of ADHD, monitoring and evaluation of adherence to clinical guidelines is important to track changes and identify where improvements are needed [27]. Currently, there is little available evidence of levels of quality of care for ADHD outside of North America; particularly across the patient journey and using a range of clinical indicators (e.g., diagnosis, treatment, ongoing management) and involving a review of medical records, rather than reliance on self-report data. Such information could provide baseline data for future healthcare system planning. In response to the need to evaluate adherence to clinical guidelines in pediatric populations in Australia, the CareTrack Kids (CTK) study was undertaken [28, 29]. CTK assessed care of Australian children (aged 0 to 15 years, in 2012 and 2013, in Queensland, New South Wales (NSW) and South Australia. CTK involved a review of medical records to determine the proportion of CPG adherence for 17 common conditions, including ADHD (28). Across the 17 conditions, guideline-adherence was estimated at 59.8% (95% Confidence Interval (CI): 57.5–62.0); the overall result for ADHD was 83.6% (95% CI: 77.7–88.5) [25]. The aim of the current paper is to describe and report indicator-level CTK results for this study.

## Methods

The CTK methods have been described in detail elsewhere [28, 29]. Here, we describe some aspects specifically relevant to ADHD.

### Development of indicators

We modified and applied the RAND-UCLA method to develop indicators [30]. For the purposes of this study, a clinical indicator was defined as a measurable component of a standard or guideline, with explicit criteria for inclusion, exclusion, time frame and practice setting [27, 31]. A systematic search for Australian and international CPGs relating to ADHD care for children relevant for the years 2012 to 2013 was undertaken [28]. Four CPGs were located: 1) the CPPs on the Diagnosis, Assessment and Management of ADHD in Children and Adolescents from the NHMRC [2]; 2) the American Academy of Pediatrics guidelines [32]; 3) a perspective published in the Australian Doctor [33]; and 4) the National Institute for Health and Care Excellence (NICE) ADHD guidelines [34]. The 2012 NHMRC CPPs, a main reference for Australian practice at the time, required that DSM-IV or ICD10 criteria be met. While DSM-5 guidelines have since become available, discussion and comparisons to standard guidelines in this manuscript will reference DSM-IV as these were applicable to the period under study. As the core symptoms of ADHD between DSM-IV and DSM-5 were unchanged [35, 36] this does not change the relevance of the indicators used in the study as a measure of guideline adherence.

Researchers with experience in clinical indicator development (LW, PDH) extracted CPG recommendations, collated expert feedback and revised the content, structure, and format of each indicator between review rounds. From the four CPGs, 43 recommendations were extracted and considered for inclusion [31]. Recommendations were excluded if they were guiding statements only with no recommended actions; if they used terms such as “may”, “consider” and “could”; if there was a low likelihood of the information being documented in the medical record; or if they were out of scope for our purposes (such as structure-level measures). This process excluded 17 recommendations, with 26 passed to the review process.

These 26 initial draft recommendations were subjected to a three-round modified Delphi “internal review” by four clinicians involved in the study (one GP and three pediatricians) followed by further reviews and modifications by four pediatricians, two psychologists, two psychiatrists, and a GP external to the project (“external review”) [31, 37]. Each reviewer recorded whether each recommendation was acceptable and feasible to collect, and its level of clinical impact, with external reviewers also scoring the appropriateness of the indicator to pediatric care in Australia in 2012 and 2013 [31]. Fourteen recommendations with low scores on these dimensions were excluded [28], leaving 12 recommendations suitable for the audit; these were formatted into 34 medical record audit indicator questions (see S1 Appendix).

### Sample size, sampling process and data collection

CTK targeted 400 medical records for ADHD and 6,000 for the 16 other conditions. Without adjustment for the design effect, a minimum of 400 visits per condition was sought to obtain national estimates with 95% Confidence Interval (CI) and precision of +/- 5% at condition level, conservatively assuming only one eligible indicator per visit. If any of the 6,400 medical records targeted and sampled contained an occasion of care for ADHD, indicator adherence was assessed. Further details on sample size and methods are provided elsewhere [28] and additional details specific to ADHD are contained in S2 Appendix. In summary, we sampled two healthcare settings for ADHD: general practices and pediatricians’ offices in randomly-selected health districts in Queensland (Hospital Health Services), NSW (Local Health Districts) and South Australia (Local Health Networks), for children aged ≤ 15 years receiving care in 2012 and 2013. Across the entire CTK study, the recruitment rate was estimated to be 24% for GPs and 25% for pediatricians.

Data were collected by eight experienced pediatric nurses (“surveyors”), trained to assess eligibility for indicator assessment and adherence to CPGs. Surveyors undertook criterion-based medical record reviews using an electronic data collection tool. They had access to extensive instructions and definitions to assist with making their decisions, including what constituted a comorbid illness. Surveyors were also instructed to not make assumptions and to rely on only the information provided in the patient’s medical record (see S3 Appendix for excerpts from surveyor manual). Medical records for selected visits in 2012 and 2013 were reviewed on-site at each participating facility during March–October 2016. Surveyors accessed the original medical records but de-identified all data for entry into a web based tool and subsequent analyses. Additional details can be found in S2 Appendix.

### Analysis

At indicator level, adherence was estimated as the percentage of indicators with ‘Yes’ or ‘No’ responses for adherence, which were scored ‘Yes’. Bundles of care were also created by aggregating related indicators into four groups which spanned the diagnosis and treatment timeline, with adherence calculated for each. For example, the assessment bundle comprising indicators ADHD04-ADHD08, would be rated as adherent only if all five of the indicators were marked ‘Yes’. When assessing bundles, a visit was only included if there were responses for all component indicators.

Sampling weights were constructed as described in S2 Appendix, to adjust for oversampling of states and healthcare settings and for sampling within health districts. Weighted data were analysed in SAS version 9.4 (SAS Institute Inc, North Carolina, USA), using the SURVEYFREQ procedure. Variance was estimated by Taylor series linearization and the primary sampling unit (health district) was specified as the clustering unit. Where appropriate, domain analysis was used. Exact 95% CIs were generated using the modified Clopper-Pearson method, except when the point estimate was 0% or 100% where the unmodified Clopper-Pearson method was used. In both indicator and bundle reports, results were suppressed if there were <25 eligible visits. P-values for differences in adherence rates between GPs and pediatricians were calculated using the Rao-Scott Chi-square test (F-test approximation), which adjusts for the design effect. Additional information can be found in S2 Appendix. Due to the varying roles of GPs and pediatricians in diagnosis and management of ADHD, overall adherence was calculated, however the majority of examinations were performed with GP and pediatrician data segregated.

### Ethical considerations

Primary ethics approval was granted by the Royal Australian College of General Practitioners’ Human Research Ethics Committee (NREEC 14–008), Women’s and Children’s Health Network of South Australia Human Research Ethics Committee (HREC/14/WCHN/68), the Sydney Children’s Health Network Human Research Ethics Committee (HREC/14/SCHN/113) and the Children’s Health Queensland Human Research Ethics Committee (HREC/14/QRCH/91), and site-specific approvals from 34 sites. Ethics approvals included reporting by healthcare setting for condition-level data. Australian human research ethics committees can waive requirements for patient consent for external access to medical records if the study entails minimal risk to facilities, clinicians, and patients; all relevant bodies provided this waiver. The study was protected from litigation by gaining statutory immunity as a quality assurance activity from the Federal Minister for Health under Part VC of the Australian Health Insurance Act 1973. Further details are reported elsewhere [28].

## Results

In total, 306 children had one or more occasions of care eligible for assessment for ADHD. Almost half the children were aged nine years or older, with three-quarters male (Table 1). There was a median of two visits per child in the sample. The surveyors conducted 6,544 eligible indicator assessments during 591 visits, at a median of 11 indicators assessed per visit. Eligible ADHD assessments were conducted in 46 GP clinics and 20 pediatrician practices.

**Table 1:** Characteristics of the eligible children, 2012–2013.

Characteristic	Children in the CTK Study (n = 306)
Age ^†^ - no. (%)	
< 4 years	4 (1.3)
4–6 years	74 (24.2)
7–8 years	82 (26.8)
9–11 years	80 (26.1)
12–15 years	66 (21.6)
Male—no. (%)	234 (76.5)

^†^ The child’s age was calculated as the age at visit where there was only one, or the midpoint of the child’s age at his first and last ADHD visit. The minimum age included was two years.

### Guideline adherence

As previously reported [29], averaged across all the indicators and practitioners, guideline adherence was 83.6% (95% CI: 77.7–88.5). Separated by practitioner type, averages were: pediatricians 90.1% (95% CI: 73.0–98.1) and GPs 68.3% (95% CI: 46.0–85.8) (Table 2, p = 0.02).

**Table 2:** Adherence by healthcare setting, 2012–2013.

Healthcare Setting	No. of Children	No. of Visits	No. of Indicators	Proportion Adherent % (95% CI)
General Practitioner	172	300	3084	68.3 (46.0, 85.8)
Pediatrician	134	291	3460	90.1 (73.0, 98.1)

Table 3 outlines adherence, by indicator separately for GPs and pediatricians. Adherence is not reported for three of the 34 indicators, because they had fewer than 25 survey assessments. For the 31 indicators where adherence was reported, adherence ranged from 26.9% for GPs (95% CI: 14.2–43.3) for indicator ADHD23 (“*Children with ADHD prescribed stimulant medication (methylphenidate and dexamphetamine sulphate) had the treatment duration and signals for stopping documented prior to prescription*.”) to 100% for pediatricians for several indicators (e.g., ADHD11: “*Children newly diagnosed with ADHD had symptoms which persisted over time (at least six months)*”; 95% CI: 94.5–100).

**Table 3:** Adherence by clinical indicator and healthcare setting, 2012–2013.

Indicator ID	Indicator Description	Healthcare Setting	No. of Children	No. of Visits	Proportion Adherent % (95% CI)
ADHD01	Children who presented to their GP with symptoms/signs of ADHD had an initial assessment documented.	GP	61	72	88.8 (75.2, 96.4)
ADHD02	Children who presented to their GP with symptoms/signs of ADHD were referred to a clinical specialist.	GP	67	84	95.5 (88.6, 98.8)
ADHD03	Parents of children who presented to their GP with symptoms/signs of ADHD were provided educational and training program information.	GP	64	80	58.5 (25.7, 86.5)
ADHD04	Children who presented to a clinical specialist with symptoms/signs of ADHD had a comprehensive medical, developmental and mental health assessment.	Pediatrician	67	70	98.4 (88.4, 100)
ADHD05	Children who presented to a clinical specialist with symptoms/signs of ADHD had a psychosocial assessment which included their family.	Pediatrician	67	70	99.2 (93.4, 100)
ADHD06	Children who presented to a clinical specialist with symptoms/signs of ADHD had a holistic assessment which included their needs, family, social and educational circumstances.	Pediatrician	67	70	98.3 (85.5, 100)
ADHD07	Children who presented to a clinical specialist with symptoms/signs of ADHD were assessed for co-existing illnesses.	Pediatrician	67	70	97.5 (86.2, 100)
ADHD08	Children who presented to a clinical specialist with symptoms/signs of ADHD were assessed for comorbid diagnosis.	Pediatrician	67	70	96.0 (80.6, 99.9)
ADHD09	Children newly diagnosed with ADHD had an onset of their symptoms in early childhood (before aged 12 years).	GP	32	35	94.3 (76.6, 99.7)
		Pediatrician	63	65	100 (94.5, 100)
		Overall	95	100	98.7 (94.0, 99.9)
ADHD10	Children newly diagnosed with ADHD showed symptoms which were maladaptive and excessive for their age and developmental level.	GP	32	32	97.8 (85.3, 100)
		Pediatrician	64	65	100 (94.5, 100)
		Overall	96	97	99.5 (95.3, 100)
ADHD11	Children newly diagnosed with ADHD had symptoms which persisted over time (at least 6 months).	GP	32	33	97.7 (85.4, 100)
		Pediatrician	64	65	100 (94.5, 100)
		Overall	96	98	99.5 (95.3, 100)
ADHD12	Children newly diagnosed with ADHD had symptoms which were evident in more than one setting.	GP	32	33	95.2 (77.9, 99.8)
		Pediatrician	64	65	100 (94.5, 100)
		Overall	96	98	98.8 (94.2, 100)
ADHD13	Children newly diagnosed with ADHD had symptoms which caused significant functional impairment.	GP	32	33	94.5 (77.7, 99.7)
		Pediatrician	64	65	97.4 (85.6, 99.9)
		Overall	96	98	96.7 (90.9, 99.2)
ADHD14	Children were diagnosed with ADHD where there was no better alternative explanation (such as another mental disorder).	GP	36	44	73.9 (22.7, 98.7)
		Pediatrician	68	82	98.8 (89.5, 100)
		Overall	104	126	92.2 (73.5, 99.2)
ADHD15	Children with ADHD had their level of impairment assessed by gathering information from multiple sources.	GP	119	201	73.8 (44.4, 92.8)
		Pediatrician	107	183	94.7 (78.0, 99.7)
		Overall	226	384	87.5 (77.8, 94.0)
ADHD16	Children with ADHD received psychological, pharmacological or educational interventions.	GP	123	209	94.4 (78.5, 99.6)
		Pediatrician	108	187	100 (98.0, 100)
		Overall	231	396	98.1 (93.9, 99.7)
ADHD17	Parents of children with ADHD were provided with information on the diagnosis and management plan.	GP	121	205	66.3 (35.6, 89.3)
		Pediatrician	106	181	94.4 (76.9, 99.7)
		Overall	227	386	84.7 (73.6, 92.5)
ADHD18	Parents of children with ADHD were advised of the potential for adverse effects of the treatment.	GP	122	207	33.1 (7.8, 68.8)
		Pediatrician	93	164	70.3 (13.7, 99.2)
		Overall	215	371	56.8 (34.2, 77.5)
ADHD19	Children with ADHD requiring medication were first prescribed a stimulant medication.	GP	41	50	76.0 (61.8, 86.9)
		Pediatrician	68	77	97.6 (91.3, 99.8)
		Overall	109	127	91.3 (79.6, 97.5)
ADHD20	Children with ADHD prescribed stimulant medication (methylphenidate and dexamphetamine sulphate) received a baseline physical assessment including, as a minimum, pulse, blood pressure, weight and height prior to prescription.	GP	37	44	63.3 (31.9, 88.1)
		Pediatrician	45	50	61.4 (40.1, 80.0)
		Overall	82	94	62.1 (45.5, 76.8)
ADHD21	Children with ADHD prescribed stimulant medication (methylphenidate and dexamphetamine sulphate) and with abnormal cardiovascular symptoms, findings or history, were referred to a cardiologist prior to prescription.	GP	4	4	Insufficient data
		Pediatrician	8	8	Insufficient data
		Overall	12	12	Insufficient data
ADHD22	Children with ADHD prescribed stimulant medication (methylphenidate and dexamphetamine sulphate) had potential harms, allergies, adverse effects and contraindications, including diversion of medications for misuse and abuse documented, prior to prescription.	GP	33	39	44.3 (17.5, 73.9)
		Pediatrician	45	50	79.8 (33.2, 99.0)
		Overall	78	89	68.6 (42.6, 88.0)
ADHD23	Children with ADHD prescribed stimulant medication (methylphenidate and dexamphetamine sulphate) had the treatment duration and signals for stopping documented prior to prescription.	GP	34	40	26.9 (14.2, 43.3)
		Pediatrician	45	48	80.7 (29.7, 99.5)
		Overall	79	88	62.1 (39.0, 81.8)
ADHD24	Children with ADHD prescribed stimulant medication had a planned schedule (follow-up, monitoring and review) documented prior to prescription.	GP	36	46	85.3 (42.7, 99.5)
		Pediatrician	46	50	97.9 (83.2, 100)
		Overall	82	96	92.9 (80.8, 98.5)
ADHD25	Children with ADHD were monitored at each visit.	GP	141	248	73.2 (40.6, 93.8)
		Pediatrician	128	271	97.4 (87.3, 99.9)
		Overall	269	519	90.7 (82.5, 95.9)
ADHD26	Children with ADHD had their management plan reviewed at least every 6 months.	GP	139	247	77.5 (55.8, 91.9)
		Pediatrician	126	271	97.9 (91.3, 99.9)
		Overall	265	518	92.2 (87.2, 95.8)
ADHD27	Children with ADHD had a management plan which was relevant to their current symptoms.	GP	138	246	83.9 (61.2, 96.2)
		Pediatrician	128	271	99.6 (96.8, 100)
		Overall	266	517	95.3 (91.0, 97.9)
ADHD28	Children with ADHD and no evidence of improvement had their stimulant medication (methylphenidate and dexamphetamine sulphate) ceased.	GP	10	14	Insufficient data
		Pediatrician	9	11	Insufficient data
		Overall	19	25	57.4 (29.3, 82.3)
ADHD29	Children with ADHD and unacceptable side effects had their stimulant medication (methylphenidate and dexamphetamine sulphate) ceased.	GP	6	6	Insufficient data
		Pediatrician	11	19	Insufficient data
		Overall	17	25	57.2 (16.5, 91.5)
ADHD30	Children with ADHD prescribed stimulant medication (methylphenidate and dexamphetamine sulphate) had their psychological symptoms and side effects assessed every 6 months.	GP	112	202	77.8 (53.8, 93.0)
		Pediatrician	94	205	98.3 (92.5, 99.9)
		Overall	206	407	92.4 (86.9, 96.1)
ADHD31	Children with ADHD prescribed stimulant medication (methylphenidate and dexamphetamine sulphate) had their growth parameters recorded every 6 months.	GP	113	203	62.0 (41.8, 79.6)
		Pediatrician	93	205	93.6 (80.2, 99.0)
		Overall	206	408	84.4 (74.8, 91.4)
ADHD32	Children with ADHD prescribed stimulant medication (methylphenidate and dexamphetamine sulphate) had their heart rate measured every 6 months.	GP	113	203	27.9 (5.4, 64.1)
		Pediatrician	93	204	51.9 (10.5, 91.3)
		Overall	206	407	45.0 (29.4, 61.2)
ADHD33	Children with ADHD prescribed stimulant medication (methylphenidate and dexamphetamine sulphate) had their blood pressure measured every 6 months.	GP	113	203	33.3 (17.5, 52.3)
		Pediatrician	93	205	70.1 (38.8, 91.7)
		Overall	206	408	59.4 (47.9, 70.2)
ADHD34	Children (aged < 7 years) with ADHD prescribed stimulant medication (methylphenidate and dexamphetamine sulphate) were assessed for adverse effects (BP, height and weight).	GP	15	21	Insufficient data
		Pediatrician	21	43	60.8 (14.5, 95.3)
		Overall	36	64	57.0 (26.5, 83.9)

GP = General Practitioner; DSM-IV = Diagnostic and Statistical Manual of Mental Disorders.

The assessed adherence of four bundles is shown in Table 4. Bundle A was restricted to children presenting to pediatricians, while bundles B to D applied to both settings. Bundle A assessed five components of appropriate assessment for children presenting with signs or symptoms of ADHD and found 95.2% adherence (95% CI: 76.6–99.9). Bundle B appraised whether children newly diagnosed with ADHD met five criteria specified in DSM-IV and found 88.3% adherence among GPs (95% CI: 55.4, 99.4) and 97.3% adherence for pediatricians (95% CI: 94.9–100); this difference was not statistically significant (p = 0.16). Bundle C assessed whether children with ADHD had three components of appropriate ongoing management and found 65.7% adherence for GPs (95% CI: 34.7–89.2) and 96.1% for pediatricians (95% CI: 83.1–99.8), with the difference being statistically significant (p = 0.0006). Bundle D assessed four components of six-monthly reviews for children with ADHD prescribed stimulant medication and found adherence of 18.7% for GPs (95% CI: 4.1–45.5) and 51.1% for pediatricians (95% CI: 9.6–91.4); the difference was not statistically significant (p = 0.07).

**Table 4:** Adherence by bundle of care, 2012–2013.

Bundle ID	Bundle Description	Indicator IDs^†^	Setting	No. of Children	No. of Visits	Proportion Adherent % (95% CI)
A	Children who presented to a clinical specialist with symptoms/signs of ADHD had appropriate assessment documented.	04–08	Pediatrician	67	70	95.2 (76.6, 99.9)
B	Children newly diagnosed with ADHD met criteria for DSM-IV.	09–13	GP	31	31	88.3 (55.4, 99.4)
			Pediatrician	63	64	97.3 (84.9, 100)
			Overall	94	95	95.2 (88.3, 98.7)
C	Children with ADHD received appropriate ongoing monitoring and management.	25–27	GP	138	244	65.7 (34.7, 89.2)
			Pediatrician	126	267	96.1 (83.1, 99.8)
			Overall	264	511	87.6 (79.2, 93.5)
D	Children with ADHD prescribed stimulant medication had a clinical assessment and review.	30–33	GP	111	201	18.7 (4.1, 45.5)
			Pediatrician	93	203	51.1 (9.6, 91.4)
			Overall	204	404	41.7 (27.5, 57.0)

DSM-IV = Diagnostic and Statistical Manual of Mental Disorders, version IV; GP = General Practitioner.

^†^In Table 3, the indicator ID number was preceded by ’ADHD’.

## Discussion

In our study, Australian children with eligible assessments for ADHD received appropriate care for an average of 83.6% (95% CI: 77.7–88.5) of indicators, which was high relative to the average across the 17 conditions from the CTK study (59.8%; 95% CI: 57.5–62.0) [28]. Indeed, in CTK, higher adherence was found for mental health conditions (>70%), in comparison to other conditions, such as tonsillitis (44%) and upper respiratory tract infection (53%). The comparatively higher results for mental health conditions, including ADHD, may in part reflect Australian initiatives over the past two decades seeking to address mental health services at a national level, such as the National Mental Health Strategy commencing in 1992, five 5-year mental health plans covering the period from 1993 to 2014, a National Action Plan of Mental Health under the Council of Australian Governments between 2006 and 2012, the Better Access initiative introduced in 2006, and the release of NHMRC guidelines in Australia on the management of ADHD [2, 38]. Such initiatives may have improved awareness among healthcare professionals regarding appropriate diagnosis and treatment of mental health conditions broadly [39]. It is also likely that healthcare practitioners are particularly scrupulous in their management of ADHD in response to the ongoing controversies surrounding its diagnosis and management, related to potential overdiagnosis and a simultaneous concern related to overprescription [40, 41].

Highest adherence was recorded for indicators that addressed assessment and short-term management of ADHD. Overall guideline adherence for Bundle A assessing components of appropriate ADHD assessment was 95.2% (95% CI: 76.6–99.9) and for Bundle B appraising whether children newly diagnosed with ADHD met DSM-IV criteria was 95.2% (95% CI: 88.3–98.7). The overall high rate of adherence to guidelines for the initial assessment, diagnosis and short-term management of ADHD suggests that, in general, these aspects of guidelines are relatively accessible, known, and feasible to implement in routine clinical practice. Further, these findings are broadly consistent with previous UK research examining healthcare professionals’ attitudes towards implementing the NICE ADHD guidelines [34] in clinical practice: that health professionals view most of the key guideline recommendations for ADHD assessment and short-term management as both important and feasible to implement [42].

Our results suggest that Australian clinicians are following recommendations to use stimulants ahead of other medications (ADHD19) with estimated adherence rates of 76.0% for GPs (95% CI: 91.8–86.9) and 97.6% for pediatricians (95% CI: 91.3, 99.8). Our data is in agreement with research from Australia [43, 44] and the USA, where stimulants are used extensively by pediatricians as a first line medication [19, 20]. However, lowest levels of adherence were found for longer term monitoring related to stimulant medication (Bundle D), supporting the findings of previous studies from Australia [44], the USA [17] and the UK [42]. Research has suggested possible barriers to long term monitoring of ADHD monitoring including: a lack of pediatric specialists, the failure of patients to attend routine follow-up appointments, a lack of resources within clinics, and the limited time of health professionals to coordinate care [42, 45], amongst others.

Although studies comparing healthcare professionals in their adherence to ADHD guidelines are limited, two studies from the USA reported that pediatricians were more likely to follow ADHD identification and management guidelines than family physicians (known as GPs in Australia) [19, 20]. In our study, GP adherence to guidelines was highest for case detection and assessment, but only moderate for short term management and lowest for longer term monitoring, particularly for recording info or regular monitoring related to medication management (i.e., indicators ADHD23, ADHD 31–33). As ADHD guidelines primarily target specialist mental health practitioners, including pediatricians, it is unsurprising that adherence would be lower among GPs. In Australia, GPs generally play a more limited role in stimulant prescription and management of ADHD, with pediatricians usually holding primary responsibility for diagnosis and ongoing medication prescription and monitoring in NSW and South Australia. Only in Queensland do GPs have a significant role in providing prescriptions for maintenance stimulant medication to children under 18 years of age [46]. However, this study was not designed or powered to detect differences between providers and states, therefore interpretation of these differences should be undertaken with caution. Further, possible reasons for lower levels of compliance for GPs may reflect the competing demands experienced by GPs for their attention and resources, and a lack of training or knowledge concerning optimal treatments [18, 25, 47]. Previous Australian research has also identified GPs’ preference to refer children with suspected ADHD to specialist services; citing diagnostic complexity, time constraints, insufficient education and training about the disorder as key reasons. Further, research on ‘gatekeeper’ settings, where GPs serve as a filter to diagnosis and treatment, have suggested evidence of both over- and under-recognition of ADHD within the same jurisdiction, and have highlighted evidence of potential GP misconceptions about ADHD and its management [48].

A previous Australian survey of 207 approved stimulant prescribers from NSW reported that pediatricians effectively monitor patients if prescribed stimulant medication, including physical assessments of weight, blood pressure and height in over 80% of cases [43]. In contrast, our results suggest that pediatricians may not be regularly monitoring (and/or documenting) heart rate and blood pressure, and that the low adherence to the recommendation of recording of heart rate (overall adherence: 45.0%; 95% CI: 29.4–61.2) and blood pressure (overall adherence: 59.4.0%; 95% CI: 47.9–70.2) may be dragging down the overall estimated adherence for Bundle D as other measures (e.g., growth parameters) were documented more frequently. It is possible that health professionals in our study measured and recorded blood pressure and heart rate using digital equipment at the same time (via automatic readings), but may not have documented this. However, the Delphi study of Hall et al. (2016) identified that while health professionals recognise the importance of regularly monitoring of patients prescribed stimulant medication (e.g., monitoring blood pressure and health rate), this was acknowledged as difficult to achieve in routine clinical practice [42].

Overall, the findings of this study suggest there is scope for improved monitoring, particularly with documenting heart rate, blood pressure and weight for children on ADHD medication. Some interventions which have been trialled to increase guideline adherence for ADHD diagnosis and management include computerised decision aids in primary practices in the USA [49, 50]. A clinical decision support tool was trialled in four practices in the USA and involved providing patient-centred, evidence-based approaches for ADHD diagnosis and management [49]. A similar approach could be transferable to the Australian context, and has been previously advocated as a strategy to help facilitate the provision of guideline-adherent care, consistency and completeness of documentation, and enable ongoing and large-scale surveillance of current practice [27].

### Strengths and limitations

The main strength of the study is that a multi-stage representative sample was taken, covering Australian states containing 60% of Australia’s children, and as such is likely generalisable to much of the unsampled population. Rather than self-report, an audit of medical records allowed an assessment of real-world practice. The audits themselves were carried out by experienced pediatric Registered Nurses who had undergone extensive training for the task. The whole medical record was available to the surveyors and guideline compliance data were collected for the two-year study period of interest (2012–2013); this means longer-term follow-up care for ADHD was not examined.

As to weaknesses, the study used documentation to assess actual practice; i.e., assuming that the action was not done if it was not documented. This may have affected results for some indicators where activities were not as readily documented (e.g., ADHD 34 which assesses monitoring of patients on stimulant medication for adverse effects). However, we argue that medical record documentation is a fundamental aspect of clinical practice and essential for delivering high quality and safe care to a population of patients [51]. We note that none of the clinical indicators used for this study stipulated the need for surveyors to enquire about the use of complementary and alternative medicines (CAMs). Research on parents reports indicate that CAMS (e.g., fish oil, chiropractor, neurofeedback, exercise therapies) are commonly used by children with ADHD [52]. Stimulant medications can have intolerable side effects (e.g., headache, nausea, and anorexia) with some parents expressing concern about “overmedicating their child”, and may opt for CAMs [53] or non-stimulatory medications (such as atomoxetine and bupropion) [54] as an alternative; thus presenting a potential source of divergence from the CPGs. However, research suggests that only 30–65% of families discuss pediatric CAM use with a doctor [55]. Doctors should inquire about the use of CAM, and use available resources to help guide families in their therapeutic choices and identify possible interactions with therapeutic drugs [55].

The study was able to examine care delivered by both GPs and community pediatricians in three states in Australia, which cover 60% of the Australian pediatric population. Other specialist groups such as mental health teams, allied health professionals, psychiatrists, and neurologists were not sampled. There is limited information in Australia on the proportion of children with ADHD seen by these practitioners. Data from the Young Minds Survey provides a breakdown of service use by children with any mental health condition, and the majority present to GPs and pediatricians [1].

However, our study is limited by self-selection bias, with sampled GPs and pediatricians possibly more confident in their practice and record keeping, which could lead to an overestimation of adherence. Moreover, the generalisability of the pediatrician findings is limited to children who could access and afford to see a pediatricians, as most pediatricians in Australia charge more than the Medicare schedule fee and require up-front full or gap payment (some children with ADHD can only access services through fully Medicare rebated hospital outpatient clinics). While we targeted a minimum of 400 medical records, we were only able to identify 306 records through our sampling, leading to wider than expected confidence intervals around our estimates of adherence for many indicators and settings.

Finally, we acknowledge that our ADHD sample was predominantly male (n = 234, 76.5%); reflecting representative population-based studies showing that the male to-female ratio of ADHD is approximately 3:1 [56]. Some research suggest that girls with ADHD are more likely than boys to have the inattentive type of ADHD and may suffer more from internalising symptoms and inattention, in contrast with the hyperactive and aggressive symptoms shown by boys. Differences in ADHD presentation between boys and girls may explain the lower prevalence rates of ADHD in females. Although this was not the focus of this research, the need for gender-specific norms and guidelines when assessing symptoms of ADHD may be warranted [57] and is an area for further investigation.

## Conclusion

To our knowledge, this is the largest cross-sectional survey of pediatric medical care for ADHD in Australia. Our methodology provides valuable baseline data on the appropriateness of care for children with ADHD, which is necessary for benchmarking purposes and the targeting and evaluation of national initiatives for specific gaps identified in this care, to be assessed by future studies. With the exception of baseline assessment and routine monitoring of heart rate and blood pressure, adherence to CPGs for ADHD by pediatricians treating patients in Australia is high. Adherence to CPGs for ADHD by GPs is lower across most domains; further research is needed to understand the reasons for GP divergence from CPGs. Timely recognition of medication side effects may be a particular area for improvement, particularly monitoring and documentation of this in medical records.

## Supporting information

S1 Appendix Characteristics, by clinical indicator, 2012–2013. Table of 34 medical record audit indicator questions.
(DOCX)

S2 Appendix Additional details relating to study methods. Details the selected methods specifically relevant to ADHD.
(DOCX)

S3 Appendix CareTrack kids surveyor manual information. Excerpt from the Surveyor manual.
(DOCX)

## Abbreviations

ADHD: Attention deficit hyperactivity disorder
CI: Confidence interval
CPG: Clinical practice guideline
CPPs: Clinical practice points
CTK: CareTrack Kids
DSM: Diagnostic and statistical manual
GP: General practitioner
NHMRC: National Health and Medical Research Council
NSW: New South Wales
USA: United States of America

## References

[1] Lawrence D, Johnson S, Hafekost J, Boterhoven De Haan K, Sawyer M, Ainley J, et al The Mental health of children and adolescents: Report on the second Australian Child and Adolescent Survey of Mental Health and Wellbeing. Canberra, AU: Department of Health, Australian Government; 2015.

[2] National Health and Medical Research Council. Clinical practice points on the diagnosis, assessment and management of attention deficit hyperactivity disorder. Canberra, AU: Australian Commonwealth Government; 2012.

[3] Sciberras E, Roos LE, Efron D. Review of prospective longitudinal studies of children with ADHD: mental health, educational, and social outcomes. Curr Atten Disord Rep. 2009;1(4): 171–177.

[4] Shaw M, Hodgkins P, Caci H, Young S, Kahle J, Woods AG, et al A systematic review and analysis of long-term outcomes in attention deficit hyperactivity disorder: effects of treatment and non-treatment. BMC Med. 2012;10(1): 99 10.1186/1741-7015-10-99 22947230 PMC3520745

[5] Hamed AM, Kauer AJ, Stevens HE. Why the diagnosis of attention deficit hyperactivity disorder matters. Front Psychiatry. 2015;6: 168 10.3389/fpsyt.2015.00168 26635643 PMC4659921

[6] Olson BG, Rosenbaum PF, Dosa NP, Roizen NJ. Improving guideline adherence for the diagnosis of ADHD in an ambulatory pediatric setting. Ambul Pediatr. 2005;5(3): 138–142. 10.1367/A04-047R.1 15913406

[7] Hinshaw SP, Scheffler RM, Fulton BD, Aase H, Banaschewski T, Cheng W, et al International variation in treatment procedures for ADHD: social context and recent trends. Psychiatr Serv. 2011;62(5): 459–464. 10.1176/ps.62.5.pss6205_0459 21532069

[8] Wasserman RC, Kelleher KJ, Bocian A, Baker A, Childs GE, Indacochea F, et al Identification of attentional and hyperactivity problems in primary care: a report from pediatric research in office settings and the ambulatory sentinel practice network. Pediatrics. 1999;103(3): e38 10.1542/peds.103.3.e38 10049994

[9] Mayne SL, Ross ME, Song L, McCarn B, Steffes J, Liu W, et al Variations in mental health diagnosis and prescribing across pediatric primary care practices. Pediatrics. 2016;137(5): e20152974 10.1542/peds.2015-2974 27244791 PMC4845867

[10] Sciutto MJ, Eisenberg M. Evaluating the evidence for and against the overdiagnosis of ADHD. J Atten Disord. 2007;11(2): 106–113. 10.1177/1087054707300094 17709814

[11] Morley CP. Disparities in ADHD assessment, diagnosis, and treatment. The International J Psychiatry Med. 2010;40(4): 383–389. 10.2190/PM.40.4.b 21391409

[12] Partridge B, Lucke J, Hall W. Over-diagnosed and over-treated: a survey of Australian public attitudes towards the acceptability of drug treatment for depression and ADHD. BMC Psychiatry. 2014;14(1): 74 10.1186/1471-244X-14-74 24625135 PMC3975148

[13] Bruchmüller K, Margraf J, Schneider S. Is ADHD diagnosed in accord with diagnostic criteria? Overdiagnosis and influence of client gender on diagnosis. J Consult Clinl Psychol. 2012;80(1): 128 10.1037/a0026582 22201328

[14] Elder TE. (2010). The importance of relative standards in ADHD diagnoses: evidence based on exact birth dates. J Health Econ. 2010;29(5): 641–656. 10.1016/j.jhealeco.2010.06.003 20638739 PMC2933294

[15] Rushton JL, Fant KE, Clark SJ. Use of practice guidelines in the primary care of children with attention-deficit/hyperactivity disorder. Pediatrics. 2004;114(1): e23–e28. 10.1542/peds.114.1.e23 15231969

[16] Chung J, Sunday S, Meryash D, Gutman A, Adesman A, editors. Medication Management of Preschool ADHD by Pediatric Sub-Specialists: Non-Compliance with AAP Clinical Guidelines. Annual meeting of the Pediatric Academic Societies, Washington, DC; 2013.

[17] Epstein JN, Kelleher KJ, Baum R, Brinkman WB, Peugh J, Gardner W, et al Variability in ADHD care in community-based pediatrics. Pediatrics. 2014;134(6): 1136–1143. 10.1542/peds.2014-1500 25367532 PMC4243070

[18] Chan E, Hopkins MR, Perrin JM, Herrerias C, Homer CJ. Diagnostic practices for attention deficit hyperactivity disorder: a national survey of primary care physicians. Ambul Pediatr. 2005;5(4): 201–208. 10.1367/A04-054R1.1 16026184

[19] McElligott JT, Lemay JR, O’Brien ES, Roland VA, Basco WT Jr, Roberts JR. Practice patterns and guideline adherence in the management of attention deficit/hyperactivity disorder. Clin Pediatr (Phila). 2014;53(10): 960–966. 10.1177/0009922814540985 24982441

[20] Wolraich ML, Bard DE, Stein MT, Rushton JL, O’Connor KG. Pediatricians’ attitudes and practices on ADHD before and after the development of ADHD pediatric practice guidelines. J Atten Disord. 2010;13(6): 563–572. 10.1177/1087054709344194 19706877

[21] Moran A, Serban N, Danielson ML, Grosse SD, Cuffe SP. Adherence to recommended care guidelines in the treatment of preschool-age Medicaid-enrolled children with a diagnosis of ADHD. Psychiatr Serv. 2019;70(1): 26–34. 10.1176/appi.ps.201800204 30373494 PMC6408287

[22] Efron D, Sciberras E, Hiscock H, Jongeling B, Lycett K, Bisset M, et al The diagnosis of attention‐deficit/hyperactivity disorder in Australian children: current paediatric practice and parent perspective. J Paediatr Child Health. 2016;52(4): 410–416. 10.1111/jpc.13091 27145504

[23] Gardner W, Kelleher KJ, Pajer K, Campo JV. Follow-up care of children identified with ADHD by primary care clinicians: a prospective cohort study. J Pediatr. 2004;145(6): 767–771. 10.1016/j.jpeds.2004.08.028 15580198

[24] Physicians RACo. Draft Australian guidelines on attention-deficit-hyperactivity/disorder. National Health and Medical Research Council Sydney; 2009.

[25] Shaw K, Wagner I, Eastwood H, Mitchell G. (2003). A qualitative study of Australian GPs’ attitudes and practices in the diagnosis and management of attention-deficit/hyperactivity disorder (ADHD). Fam Pract. 2003;20(2): 129–134. 10.1093/fampra/20.2.129 12651785

[26] Hiscock H, Danchin MH, Efron D, Gulenc A, Hearps S, Freed GL, et al Trends in paediatric practice in Australia: 2008 and 2013 national audits from the Australian Paediatric Research Network. J Paediatr Child Health. 2017;53(1): 55–61. 10.1111/jpc.13280 27594610

[27] Runciman WB, Coiera EW, Day RO, Hannaford NA, Hibbert PD, Hunt TD, et al Towards the delivery of appropriate health care in Australia. Med J Aust. 2012;197(2): 78–81. 10.5694/mja12.10799 22794043

[28] Braithwaite J, Hibbert PD, Jaffe A, White L, Cowell CT, Harris MF, et al Quality of health care for children in Australia, 2012–2013. JAMA. 2018;319(11): 1113–1124. 10.1001/jama.2018.0162 29558552 PMC5885883

[29] Hooper TD, Hibbert PD, Mealing N, Wiles L, Jaffe A, White L, et al CareTrack Kids—part 2. Assessing the appropriateness of the healthcare delivered to Australian children: study protocol for a retrospective medical record review. BMJ Open. 2015;5(4): e007749 10.1136/bmjopen-2015-007749 25854977 PMC4390725

[30] Fitch K, Bernstein SJ, Aguilar MD, Burnand B, LaCalle JR. The RAND/UCLA appropriateness method user’s manual. Santa Monica, CA: RAND Corporation; 2001.

[31] Wiles L, Hooper TD, Hibbert PD, Molloy C, White L, Jaffe A, et al Clinical indicators for common paediatric conditions: processes, provenance and products of the CareTrack Kids study. PLoS One. 2019;14(1): e0209637 10.1371/journal.pone.0209637 30625190 PMC6326465

[32] Subcommittee on Attention-Deficit/Hyperactivity Disorder, Steering Committee on Quality Improvement and Management. ADHD: Clinical Practice Guideline for the diagnosis, evaluation, and treatment of attention-deficit/hyperactivity disorder in children and adolescents. Pediatrics. 2011;128(5): 1007–1022. 10.1542/peds.2011-2654 22003063 PMC4500647

[33] Kohn M, White R. How to treat: child and adolescent ADHD. Australian Doctor. 2008;28(Nov): 25–32.

[34] National Institute for Health and Care Excellence. Attention deficit hyperactivity disorder: diagnosis and management (CG72). London, UK: NICE; 2008. 29634174

[35] American Psychiatric Association. Diagnostic and statistical manual of mental disorders. Fifth ed Arlington, VA: American Psychiatric Publishing; 2013.

[36] Epstein JN, Loren REA. Changes in the definition of ADHD in DSM-5: subtle but important. Neuropsychiatry. 2013;3(5): 455–458. 10.2217/npy.13.59 24644516 PMC3955126

[37] Wiles L, Hooper TD, Hibbert PD, White L, Mealing N, Jaffe A, et al CareTrack Kids—part 1. Assessing the appropriateness of healthcare delivered to Australian children: study protocol for clinical indicator development. BMJ Open. 2015;5(4): e007748 10.1136/bmjopen-2015-007748 25854976 PMC4390723

[38] Royal Australasian College of Physicians. Draft Australian guidelines on attention deficit hyperactivity disorder (ADHD). Canberra, ACT: NHMRC; 2009.

[39] Pirkis J, Harris M, Hall W, Ftanou M. Evaluation of the better access to psychiatrists, psychologists and general practitioners through the Medicare benefits schedule initiative. Melbourne, AU: Centre for Health Policy Programs and Economics, University of Melbourne; 2011.

[40] Dunlop AJ, Newman LK. ADHD and psychostimulants—overdiagnosis and overprescription. Med J Aust. 2016;204(4): 139–140. 10.5694/mja15.01387 26937659

[41] Rollins A. ADHD under-recognised, not over-diagnosed: Expert Aust Med. 2014;26(16): 15–16.

[42] Hall CL, Taylor JA, Newell K, Baldwin L, Sayal K, Hollis C. The challenges of implementing ADHD clinical guidelines and research best evidence in routine clinical care settings: Delphi survey and mixed-methods study. B J Psych Open. 2016;2(1): 25–31. 10.1192/bjpo.bp.115.002386 27703750 PMC4995556

[43] Mitchell PB, Levy F, Hadzi‐Pavlovic D, Concannon PE, Hutchins P, Mulcahy DL, et al Practitioner characteristics and the treatment of children and adolescents with attention deficit hyperactivity disorder. J Paediatr Child Health. 2012;48(6): 483–489. 10.1111/j.1440-1754.2011.02242.x 22111981

[44] Hazell PL, Walton JM, McDowell MJ. Management of children prescribed psychostimulant medication for attention deficit hyperactivity disorder in the Hunter region of NSW. Med J Aust. 1996;165(9): 477–480. 10.5694/j.1326-5377.1996.tb138611.x 8937367

[45] Leslie LK, Stallone KA, Weckerly J, McDaniel AL, Monn A. Implementing ADHD guidelines in primary care: does one size fit all? J Health Care Poor Underserved. 2006;17(2): 302–327. 10.1353/hpu.2006.0064 16702717

[46] Australian ADHD Professionals Association (AADPA). ADHD stimulant prescribing regulations & authorities in Australia & New Zealand. AADPA; 2020. [Cited 2020 November 18]. https://aadpa.com.au/adhd-stimulant-prescribing-regulations-authorities-in-australia-new-zealand/.

[47] Wang PS, Demler O, Kessler RC. Adequacy of treatment for serious mental illness in the United States. Am J Public Health. 2002;92(1): 92–98. 10.2105/ajph.92.1.92 11772769 PMC1447396

[48] Tatlow-Golden M, Prihodova L, Gavin B, Cullen W, McNicholas F. (2016). What do general practitioners know about ADHD? Attitudes and knowledge among first-contact gatekeepers: systematic narrative review. BMC Fam Pract. 2016;17(1): 129 10.1186/s12875-016-0516-x 27605006 PMC5013633

[49] Carroll AE, Bauer NS, Dugan TM, Anand V, Saha C, Downs SM. Use of a computerized decision aid for ADHD diagnosis: a randomized controlled trial. Pediatrics. 2013;132(3): e623–e629. 10.1542/peds.2013-0933 23958768 PMC3876764

[50] Johnson SA, Poon EG, Fiskio J, Rao SR, Van Cleave J, Perrin JM, et al Electronic health record decision support and quality of care for children with ADHD. Pediatrics. 2010;126(2): 239–246. 10.1542/peds.2009-0710 20643719

[51] Mann R, Williams J. Standards in medical record keeping. Clin Med (Lond). 2003;3(4): 329–332. 10.7861/clinmedicine.3-4-329 12938746 PMC5351947

[52] Efron D, Sciberras E, Hiscock H, Jongeling B, Lycett K, Bisset M, et al The diagnosis of attention‐deficit/hyperactivity disorder in Australian children: current paediatric practice and parent perspective. J Paediatr Child Health. 2016;52(4): 410–416. 10.1111/jpc.13091 27145504

[53] Ng QX, Ho CYX, Chan HW, Yong BZJ, Yeo WS. Managing childhood and adolescent attention-deficit/hyperactivity disorder (ADHD) with exercise: a systematic review. Complement Ther Med. 2017;34: 123–128. 10.1016/j.ctim.2017.08.018 28917364

[54] Stuhec M, Munda B, Svab V, Locatelli I. Comparative efficacy and acceptability of atomoxetine, lisdexamfetamine, bupropion and methylphenidate in treatment of attention deficit hyperactivity disorder in children and adolescents: a meta-analysis with focus on bupropion. J Affect Disord. 2015;178: 149–159. 10.1016/j.jad.2015.03.006 25813457

[55] Mazhar H, Harkin EF, Foster BC, Harris CS. Complementary and alternative medicine use in pediatric Attention-Deficit Hyperactivity Disorder (ADHD): reviewing the safety and efficacy of herbal medicines. Curr Dev Discord Rep. 2016;3: 15–24.

[56] Willcutt EG. The prevalence of DSM-IV attention-deficit/hyperactivity disorder: a meta-analytic review. Neurotherapeutics. 2012;9(3): 490–499. 10.1007/s13311-012-0135-8 22976615 PMC3441936

[57] Slobodin O, Davidovitch M. (2019). Gender differences in objective and subjective measures of ADHD among clinic-referred children. Front Hum Neurosci. 2019;13: 441 10.3389/fnhum.2019.00441 31920599 PMC6923191

