# Supplementary material for: Guideline adherence in the management of attention deficit hyperactivity disorder in children: An audit of selected medical records in three Australian states
Source: PLoS One. 2021 Feb 8;16(2):e0245916. doi: 10.1371/journal.pone.0245916 (PMC7869992; doi:10.1371/journal.pone.0245916)
Supplement: S1 Appendix — Table of 34 medical record audit indicator questions. (DOCX) [file pone.0245916.s001.docx]

**S1 Appendix: Characteristics, by clinical indicator, 2012 - 2013**

|  | | | **No. of Sites** | |  | | |
| --- | --- | --- | --- | --- | --- | --- | --- |
| **Indicator ID** | **Indicator Description** | **Age Inclusion Criteria** | **GP** | **P** | **Strength of recommendation^#^** | **Phase of Care** | **Quality Type*** |
| ADHD01 | Children who presented to their GP with symptoms/signs of ADHD had an initial assessment documented. | 2 - 15 years | 31 | NA | Grade B | Diagnosis | Underuse |
| ADHD02 | Children who presented to their GP with symptoms/signs of ADHD were referred to a clinical specialist. | 2 - 15 years | 33 | NA | Grade B | Diagnosis | Underuse |
| ADHD03 | Parents of children who presented to their GP with symptoms/signs of ADHD were provided educational and training program information. | 2 - 15 years | 32 | NA | Grade B | Diagnosis | Underuse |
| ADHD04 | Children who presented to a clinical specialist with symptoms/signs of ADHD had a comprehensive medical, developmental and mental health assessment. | 2 - 15 years | NA | 18 | Consensus-based recommendation | Diagnosis | Underuse |
| ADHD05 | Children who presented to a clinical specialist with symptoms/signs of ADHD had a psychosocial assessment which included their family. | 2 - 15 years | NA | 18 | Consensus-based recommendation | Diagnosis | Underuse |
| ADHD06 | Children who presented to a clinical specialist with symptoms/signs of ADHD had a holistic assessment which included their needs, family, social and educational circumstances. | 2 - 15 years | NA | 18 | Consensus-based recommendation | Diagnosis | Underuse |
| ADHD07 | Children who presented to a clinical specialist with symptoms/signs of ADHD were assessed for co-existing illnesses. | 2 - 15 years | NA | 18 | Consensus-based recommendation | Diagnosis | Underuse |
| ADHD08 | Children who presented to a clinical specialist with symptoms/signs of ADHD were assessed for comorbid diagnosis. | 2 - 15 years | NA | 18 | Consensus-based recommendation | Diagnosis | Underuse |
| ADHD09 | Children newly diagnosed with ADHD had an onset of their symptoms in early childhood (before aged 12 years). | 2 - 15 years | 17 | 17 | Consensus-based recommendation | Diagnosis | Underuse |
| ADHD10 | Children newly diagnosed with ADHD showed symptoms which were maladaptive and excessive for their age and developmental level. | 2 - 15 years | 17 | 17 | Consensus-based recommendation | Diagnosis | Underuse |
| ADHD11 | Children newly diagnosed with ADHD had symptoms which persisted over time (at least 6 months). | 2 - 15 years | 18 | 17 | Consensus-based recommendation | Diagnosis | Underuse |
| ADHD12 | Children newly diagnosed with ADHD had symptoms which were evident in more than one setting. | 2 - 15 years | 17 | 17 | Consensus-based recommendation | Diagnosis | Underuse |
| ADHD13 | Children newly diagnosed with ADHD had symptoms which caused significant functional impairment. | 2 - 15 years | 17 | 17 | Consensus-based recommendation | Diagnosis | Underuse |
| ADHD14 | Children were diagnosed with ADHD where there was no better alternative explanation (such as another mental disorder). | 2 - 15 years | 19 | 17 | Consensus-based recommendation | Diagnosis | Underuse |
| ADHD15 | Children with ADHD had their level of impairment assessed by gathering information from multiple sources. | 2 - 15 years | 34 | 19 | Consensus-based recommendation | Diagnosis | Underuse |
| ADHD16 | Children with ADHD received psychological, pharmacological or educational interventions. | 2 - 15 years | 34 | 19 | Consensus-based recommendation | Treatment | Underuse |
| ADHD17 | Parents of children with ADHD were provided with information on the diagnosis and management plan. | 2 - 15 years | 33 | 19 | Consensus-based recommendation | Treatment | Underuse |
| ADHD18 | Parents of children with ADHD were advised of the potential for adverse effects of the treatment. | 2 - 15 years | 34 | 18 | Consensus-based recommendation | Treatment | Underuse |
| ADHD19 | Children with ADHD requiring medication were first prescribed a stimulant medication. | 2 - 15 years | 18 | 15 | Grade A | Treatment | Underuse |
| ADHD20 | Children with ADHD prescribed stimulant medication (methylphenidate and dexamphetamine sulphate) received a baseline physical assessment including, as a minimum, pulse, blood pressure, weight and height prior to prescription. | 2 - 15 years | 19 | 15 | Consensus-based recommendation | Treatment | Underuse |
| ADHD21 | Children with ADHD prescribed stimulant medication (methylphenidate and dexamphetamine sulphate) and with abnormal cardiovascular symptoms, findings or history, were referred to a cardiologist prior to prescription. | 2 - 15 years | 4 | 4 | Consensus-based recommendation | Treatment | Underuse |
| ADHD22 | Children with ADHD prescribed stimulant medication (methylphenidate and dexamphetamine sulphate) had potential harms, allergies, adverse effects and contraindications, including diversion of medications for misuse and abuse documented, prior to prescription. | 2 - 15 years | 17 | 15 | Consensus-based recommendation | Treatment | Underuse |
| ADHD23 | Children with ADHD prescribed stimulant medication (methylphenidate and dexamphetamine sulphate) had the treatment duration and signals for stopping documented prior to prescription. | 2 - 15 years | 17 | 15 | Consensus-based recommendation | Treatment | Underuse |
| ADHD24 | Children with ADHD prescribed stimulant medication had a planned schedule (follow-up, monitoring and review) documented prior to prescription. | 2 - 15 years | 18 | 15 | Consensus-based recommendation | Treatment | Underuse |
| ADHD25 | Children with ADHD were monitored at each visit. | 2 - 15 years | 36 | 19 | Consensus-based recommendation | Ongoing management | Underuse |
| ADHD26 | Children with ADHD had their management plan reviewed at least every 6 months. | 2 - 15 years | 35 | 19 | Consensus-based recommendation | Ongoing management | Underuse |
| ADHD27 | Children with ADHD had a management plan which was relevant to their current symptoms. | 2 - 15 years | 35 | 19 | Consensus-based recommendation | Ongoing management | Underuse |
| ADHD28 | Children with ADHD and no evidence of improvement had their stimulant medication (methylphenidate and dexamphetamine sulphate) ceased. | 2 - 15 years | 8 | 7 | Consensus-based recommendation | Ongoing management | Underuse |
| ADHD29 | Children with ADHD and unacceptable side effects had their stimulant medication (methylphenidate and dexamphetamine sulphate) ceased. | 2 - 15 years | 6 | 8 | Consensus-based recommendation | Ongoing management | Underuse |
| ADHD30 | Children with ADHD prescribed stimulant medication (methylphenidate and dexamphetamine sulphate) had their psychological symptoms and side effects assessed every 6 months. | 2 - 15 years | 34 | 18 | Consensus-based recommendation | Ongoing management | Underuse |
| ADHD31 | Children with ADHD prescribed stimulant medication (methylphenidate and dexamphetamine sulphate) had their growth parameters recorded every 6 months. | 2 - 15 years | 34 | 18 | Consensus-based recommendation | Ongoing management | Underuse |
| ADHD32 | Children with ADHD prescribed stimulant medication (methylphenidate and dexamphetamine sulphate) had their heart rate measured every 6 months. | 2 - 15 years | 34 | 18 | Consensus-based recommendation | Ongoing management | Underuse |
| ADHD33 | Children with ADHD prescribed stimulant medication (methylphenidate and dexamphetamine sulphate) had their blood pressure measured every 6 months. | 2 - 15 years | 34 | 18 | Consensus-based recommendation | Ongoing management | Underuse |
| ADHD34 | Children (aged < 7 years) with ADHD prescribed stimulant medication (methylphenidate and dexamphetamine sulphate) were assessed for adverse effects (blood pressure, height and weight). | 2 - 6 years | 12 | 8 | Consensus-based recommendation | Ongoing management | Underuse |

Legend: ID=Identifier; GP=General Practitioner; P=Pediatrician; DSM-IV=Diagnostic and Statistical Manual of Mental Disorders.

^#^ Strength of recommendation as reported in individual CPGs. CPGs used a variety of classification schemes for allocating Strength of Recommendation in Grades (with Grade A indicating the strongest recommendation in all classification schemes). If strength of recommendation, or Level of Evidence, were not specified in the CPG, the term “Consensus-based recommendation” was assigned.

* The type of quality of care assessed was classified as underuse or overuse: underuse refers to actions which are recommended, but not undertaken; overuse refers to actions which are not indicated, or are contraindicated, in the context of the indicator’s inclusion criteria.
