# Supplementary material for: Guideline adherence in the management of attention deficit hyperactivity disorder in children: An audit of selected medical records in three Australian states
Source: PLoS One. 2021 Feb 8;16(2):e0245916. doi: 10.1371/journal.pone.0245916 (PMC7869992; doi:10.1371/journal.pone.0245916)
Supplement: S2 Appendix — Details the selected methods specifically relevant to ADHD. (DOCX) [file pone.0245916.s002.docx]

# **S2 Appendix: Additional details relating to study methods**

The report of top-level CareTrack Kids (CTK) results.(Braithwaite et al., 2018) and its associated online appendix, detail the methods of the larger study, which generated the data reported in this paper. Selected methods specifically relevant to ADHD are described below.

**Sample size**

A visit was defined as a consultation with either a General Practitioner (GP) or a paediatrician. Without adjustment for the design effect, a minimum of 400 visits per condition was required to obtain national estimates with 95% Confidence Interval (CI) and precision of +/- 5% at condition level, conservatively assuming only one eligible indicator per visit. It was anticipated that loss of precision due to design effects would be largely offset by multiple eligible indicators per visit and additional visits generated by the secondary sampling (multiple visits for care of ADHD for each medical record identified for sampling of ADHD, and visits for care of ADHD incidentally found in medical records identified for sampling other conditions).

**Sampling** **Process**

A multistage stratified random sampling process was implemented. For logistical efficiency, sampling was targeted at three states, Queensland (QLD), New South Wales (NSW) and South Australia (SA), which together comprise 60.0% of the estimated Australian population aged 15 years or younger in the 2012 and 2013 calendar years. State Departments of Health organise care within administrative units (‘health districts’): Hospital Health Services in QLD, Local Health Districts in NSW, and Local Health Networks in SA. For QLD, we targeted five health districts (two metropolitan, three regional), in NSW four health districts (two metropolitan, two regional), and in SA three health districts (two metropolitan, one regional). Despite best efforts, paediatricians were not recruited in targeted health districts in SA, so they were recruited in a metropolitan health district that was not randomly selected.

**Recruitment of health care providers**

Within the selected health districts, we advertised the study to general practices and paediatricians, and approached all the providers we could identify through internet searches, and via personal contacts. Within the selected sites, we sampled medical records for each condition targeted at that setting.

Recruitment of GPs and paediatricians was decentralised. Administrative details for refusal rates, from cold-calling or direct contact by clinicians who facilitated recruitment of their peers, were maintained on project laptops. At the end of recruitment all computers were decommissioned and cleaned, with the files archived on a USB. Unfortunately, the USBs created during laptop decommissioning were misplaced and have not been able to be located. This did not affect the quality indicator adherence data, as the database was remotely located and updated regularly via the internet. We have therefore sought to estimate the recruitment rates based on recruitment spreadsheets emailed to the administrative staff.

For GPs, we were only able to locate emailed spreadsheets with late stage records for one state, South Australia. Based on this spreadsheet, we approached 114 GPs and recruited 27 of them, giving a recruitment rate of 23.7%; an additional GP, not listed on the available spreadsheet, was recruited subsequently and was not added to either the numerator or the denominator, for this estimate. The spreadsheet did not have clear information on eligibility, so it is likely that an unknown number of the 114 approached were ineligible because: 1) they were not open during the whole 2012-2013 survey period; 2) they saw no or few children; or 3) they were not confident in their ability to generate full listings of children with the target conditions, or they did not use one of the four practice software systems our surveyors were trained to search. Our estimate of 23.7% is therefore likely to be an underestimate of the actual recruitment rate.

For paediatricians, we were fortunate to be able to locate emailed records with late stage records for all three states. Based on these spreadsheets, we successfully approached 80 eligible paediatricians and recruited 20 of them, giving a recruitment rate of 25.0%.

Self-selection of GPs and paediatricians, and the estimated 24-25% recruitment rate, could lead to bias in the estimated guideline adherence, arising from self-selection. It is plausible that self-selected practices were more confident of their guideline adherence, potentially leading to overestimation of the quality of care in the CareTrack Kids study.

**Allocation of visits to sampling units**

The number of ADHD records targeted at each site was determined by a nominal allocation of the 400 records targeted, informed by data available at the time, supplemented by expert opinion, with planned over-sampling of settings where fewer occasions of care were expected.(Hooper et al., 2015; Braithwaite et al., 2018) For GPs, different combinations of conditions were targeted at each site, to simplify the logistics of sampling; thus ADHD was not targeted at all participating sites. ADHD records were targeted at all paediatricians’ offices.

**Data collection**

Nine experienced paediatric nurses were employed across the three states, with eight assessing occasions of care for ADHD. The surveyors undertook a one-week training program, prior to data collection. A surveyor manual was developed which included instructions, condition-specific definitions, inclusion and exclusion criteria, and guidance for assessing eligibility of each encounter for relevant indicators.

A web-based tool, originally developed for the CareTrack Adults study,(Hunt et al., 2012; Runciman et al., 2012) was designed to enter data during medical record review. Algorithms to filter indicators by setting, and by age, were embedded in the tool. Three of the indicators were restricted to GPs, five to SPs, and 26 applied to both; one indicator was restricted to children aged < 7 years.

Surveyors undertook criterion-based medical record reviews using the data collection tool. Medical records for selected visits in 2012 and 2013 were reviewed on-site at each participating facility during March–October 2016. The surveyors responded to each indicator as ‘Yes’ (care provided during the encounter was consistent with the indicator), ‘No’, or ‘Not Applicable’ (NA; the indicator was not eligible for assessment). For example, a surveyor assessing a paediatrician visit for ongoing management of ADHD medication, would answer ‘NA’ to indicators ADHD09-ADHD13, all of which apply to new diagnoses of ADHD.

**Analysis**

Survey or register-derived data were used to estimate the proportion of occasions of care for ADHD.(Britt et al., 2013; Harrison, 2017; Hiscock et al., 2016; Hiscock, 2017) The number of occasions of healthcare for each condition was thereby estimated for each health district, or the state as a whole in the case of paediatrician visits in SA, and sampling weights were calculated using the methods detailed in eAppendix 4 of the report of the top-line CTK results (this Appendix can be accessed by request via the corresponding author, if required).(Braithwaite et al., 2018) Exact 95% CIs were generated using the modified Clopper-Pearson method, except when the point estimate was 0% or 100% where the unmodified Clopper-Pearson method was used.(Korn and Graubard, 1998)

A variety of stratifications, and sometimes domain analysis,(Lohr, 2009; Heeringa et al., 2010) were necessary to ensure accuracy of the confidence interval estimates. These are detailed in eTable 1, below.

**eTable 1: Domain analysis and stratifications for different estimates presented in the manuscript**

| Location | Sub-section/Area | Domain analysis(Lohr, 2009; Heeringa et al., 2010) | Strata |
| --- | --- | --- | --- |
| Table 1 | Indicator x healthcare setting estimates | Yes | None |
| Table 3 | Healthcare setting estimates | No | None |
| Table 4 | Bundle x healthcare setting estimates | Yes | None |

**References:**

Braithwaite J, Hibbert PD, Jaffe A, et al. (2018) Quality of health care for children in Australia, 2012-2013. *JAMA* 319: 1113-1124.

Britt H, Miller GC, Henderson J, et al. (2013) General Practice Activity in Australia 2012-13: BEACH: Bettering the Evaluation and Care of Health. Sydney: Sydney University Press.

Harrison C. (2017) BEACH 2012-13 weighted data on frequency of management of selected conditions, for children aged 0-15, by general practitioners. [Personal communication] Sydney: Menzies Centre for Health Policy, School of Public Health, The University of Sydney.

Heeringa SG, West BT and Berglund PA. (2010) Applied survey data analysis. Boca Raton: CRC Press.

Hiscock H. (2017) CAP 2013 data on frequency of management of selected conditions, for children aged 0-15, by Paediatricians. [Personal communication] Melbourne: Australian Paediatric Research Network.

Hiscock H, Danchin MH, Efron D, et al. (2016) Trends in paediatric practice in Australia: 2008 and 2013 national audits from the Australian Paediatric Research Network. *J Paediatr Child Health* 53: 55-61.

Hooper TD, Hibbert PD, Mealing N, et al. (2015) CareTrack Kids-part 2. Assessing the appropriateness of the healthcare delivered to Australian children: study protocol for a retrospective medical record review. *BMJ Open* 5: e007749.

Hunt TD, Ramanathan SA, Hannaford NA, et al. (2012) CareTrack Australia: assessing the appropriateness of adult healthcare: protocol for a retrospective medical record review. *BMJ Open* 2: e000665.

Korn EL and Graubard BI. (1998) Confidence intervals for proportions with small expected number of positive counts estimated from survey data. *Surv Methodol* 24: 193-201.

Lohr S. (2009) Sampling: design and analysis. Second ed. Boston: Brooks-Cole Publishing.

Runciman WB, Hunt TD, Hannaford NA, et al. (2012) CareTrack: assessing the appropriateness of health care delivery in Australia. *Medical Journal of Australia* 197: 100-105.
