# Supplementary material for: Guideline adherence in the management of attention deficit hyperactivity disorder in children: An audit of selected medical records in three Australian states
Source: PLoS One. 2021 Feb 8;16(2):e0245916. doi: 10.1371/journal.pone.0245916 (PMC7869992; doi:10.1371/journal.pone.0245916)
Supplement: S3 Appendix — Excerpt from the Surveyor manual. (DOCX) [file pone.0245916.s003.docx]

**S3 Appendix: CareTrack Kids Surveyor Manual information**

**Completing the CareTrack Kids indicator questions (data collection)**

Once you have entered the CTK condition dates, you will be able complete the indicator questions for each visit. When completing the CTK Db questions you need to consider the following:

1. **Inclusion Criteria:** “Do the patient’s characteristics and type of visit meet the inclusion criteria for the indicator question”? If the answer to this is no, then this would be scored as *NA*. If the answer is yes, then proceed to consideration number 2. If you answer *NA*, it will be possible for you to electronically record a reason for this in the Comments field.
2. **Compliance:** If the indicator question is relevant (i.e. meets the inclusion criteria) then you must ask yourself “Has the compliance for this indicator been met?” If it has, it will be scored *YES*; if it has not, then it will be scored *NO*. If you answer *NO*, we strongly recommended you record a reason for this (e.g. a review appointment was not scheduled, amoxicillin was prescribed).

Figure 1 shows the decision making flowchart for answering the indicator questions. In order to assist in the differentiation between inclusion criteria and compliance, in the list of indicator questions in this manual (Appendix 1) all inclusion criteria have been coloured in red and all compliance in yellow.


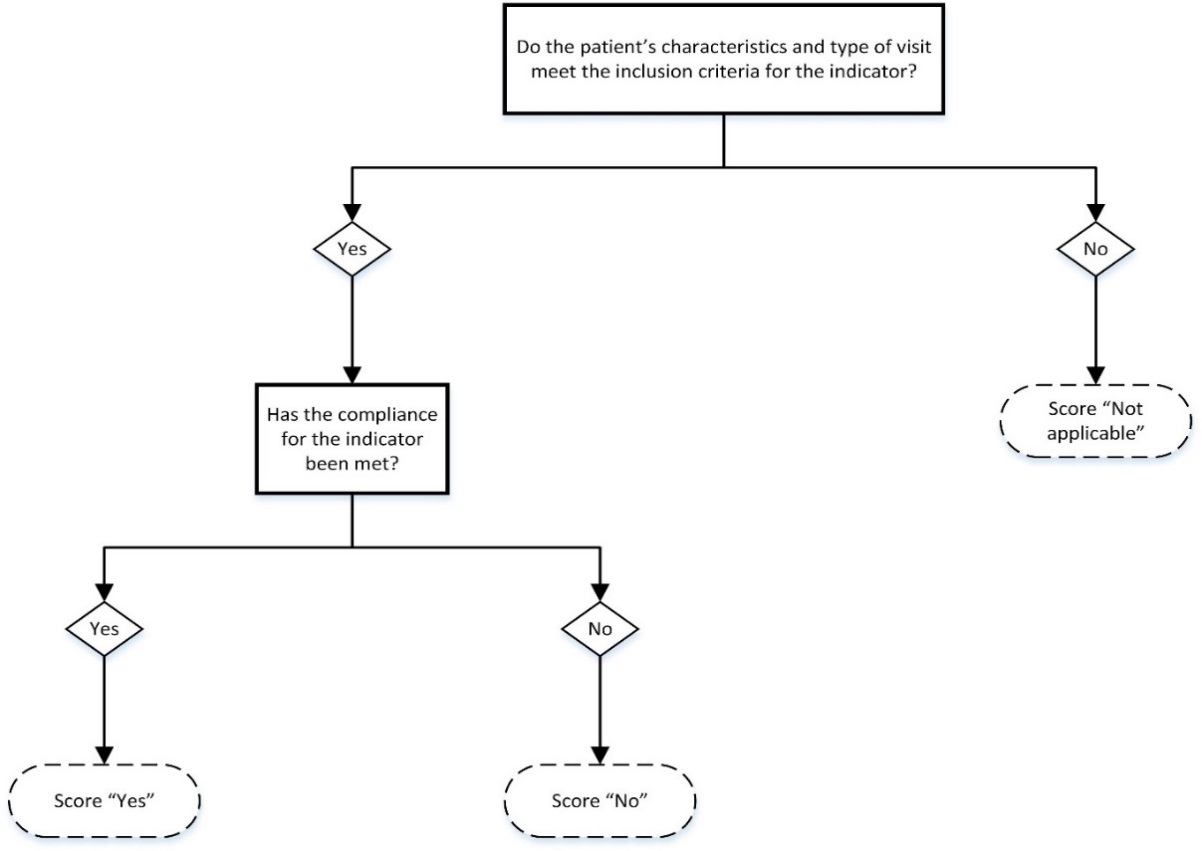


Note:

NA can only be used if the medical record scenario does not fit the inclusion criteria *(consideration A)*

If you have proceeded to the second consideration (*consideration B*), then only the scores of **Y** or **N** can be given

*Figure 1: Decision making flowchart for database questions.*

**General rules**

1. Before commencing each medical record review, please check:
   1. Patient identification – is this the correct patient? (patient ID, gender and date of birth)
   2. Condition listed in the medical record – does this match the condition the patient was selected for?
   3. Date of medical record visit – is this during the study period, i.e. 2012 -2013?
   4. The point of care / contact for each condition and healthcare practitioner (HCP) – is this the first encounter for this condition and presentation to this HCP? Does this visit correlate with diagnosis of the condition, or is this a follow-up visit?
2. Screen the entire medical record for additional sources of information, relevant for the time period:
   1. History – e.g. past medical, family/social
   2. Allergies – e.g. drug allergies, reactions to topical agents
   3. Test results - e.g. ABGs, blood tests, radiology, letters and other correspondence
3. Read indicator carefully. Pay particular attention to:
   1. Inclusion criteria – shaded in red
   2. Compliance action(s) – shaded in yellow
   3. Any definitions
   4. Get a sense of the “spirit” or intent of the indicator – e.g. exactly what is being asked? Is it assessing under-use or over-servicing (NOTE: most indicators assess under-use; over-servicing type indicators are marked with an *)?
4. If it’s not written down, it did not happen. Do not make assumptions.
5. In the following circumstances, documentation by exclusion is sufficient for compliance (e.g. HCPs do not need to re-record this information at each visit):
   1. History – e.g. past medical, family/social
   2. Allergies – e.g. drug allergies, reactions to topical agents
   3. Test results - e.g. ABGs, blood tests, radiology, letters and other correspondence
6. All indicators that relate to care provided “at diagnosis” are assessed at the first visit/encounter for this condition, at each HCP - e.g. first visit to the GP AND first visit to the specialist(s)
7. All indicators that relate to “first-line treatment” can only ever occur ONCE for each patient – e.g. if reviewing a specialists’ record, and patient has already been provided with treatment from a different HCP for this same condition, this will be marked as NA
8. All indicators with reference to a timeframe, please consider this a minimum requirement unless otherwise stated – e.g. “are reviewed every 6 months”, means that we are assessing compliance for at least one review occurring within the 6 months.
9. In the absence of definitions, use your clinical judgement to determine what is appropriate and practical to occur, and note this information in the “Comments” field (e.g. Medical record states there is no past medical history (i.e. nil) – which may not be updated at each visit, however a recent new illness should be documented at this visit)
10. If there are multiple visits in a short period of time – it will be your responsibility to use your clinical judgment to determine if it is reasonable to expect the HCP to complete all care again. In the case of some conditions or indicators it may not be appropriate. For example if a patient visited an ED repeatedly in a small timeframe (i.e. 5, 6 and 7^th^ June 2012) for anxiety - it may be appropriate for indicators such as “Children who presented with suspected anxiety had their family circumstances assessed” for the HCP to have written in the clinical notes “as per visit on 5/6”.
11. The presenting condition is audited for each visit. However if the diagnosis changes (i.e presented with Abdominal Pain and part-way through the visit is diagnosed with Acute Gastroenteritis) then from that point of time onwards, all questions for abdominal pain become NA. If the new diagnosis is a CTK condition and care is continuing at the same HCP, the surveyor will then edit visit dates and add a new visit in from that point for Acute Gastroenteritis.
12. Ideally, compliance for “monitored” will require both an initial (baseline) assessment and review over time (which, depending on the condition indicators, may be periodical or within a stipulated timeframe). However, there may be some instances where one of the other of these two criteria are not possible or appropriate, or may not be specifically recorded as such (e.g. review of treatment goals, or management). In these instances, you will need to use your clinical judgement to score the indicators. Importantly, populating the “Comments” field with relevant information about what has been documented and your decision-making process, will assist us with ongoing quality control and final data analysis.

**7.3 Condition specific definitions: ADHD**

**Active involvement:** Ongoing involvement – most likely assessed over a period of 6-12 months.

**Co-existing illnesses:** includes obstructive sleep apnoea, iron deficiency.

**Comorbid illnesses:** e.g. obesity, cardiac and lung disease

**Comprehensive medical**: includes assessment for co-existing illnesses such as obstructive sleep apnoea, hearing loss and seizure disorder.

**Developmental/mental health assessment:** includes citing of evidence of IQ tests and school reports.

**Holistic assessment:** Covering multiple domains such as family, school, social, psychosocial including strengths and vulnerabilities

**Initial assessment:** considers behavioural, emotional or cognitive symptoms causing significant and persistent impairment to a child, their family or at school.

**Multiple sources:** Refers to documentation of contact (phone, electronic or letter) with the child and parent / guardian and at least one person outside the family unit (allied health, GP, Teacher).

**Psychosocial assessment:** Social history and family dynamics should be discussed (this would include the contribution of family and social adversity, including neglect and abuse).

**Symptoms and signs of ADHD:** ADHD in children and adolescents is characterised by excessive levels of hyperactive, impulsive and inattentive behaviour. Not all children and adolescents diagnosed with ADHD will function abnormally in all three domains. Some children/adolescents are predominantly hyperactive and impulsive, while others are mainly inattentive.[[1](#_ENREF_1)]

**Stimulant medication:** If written as Ritalin and not Ritalin LA use tablet dose recommendations.

| Drug Name | Age | Dose |
| --- | --- | --- |
| Methylphenidate:  Ritalin LA Capsules  Ritalin Tablets  Concerta | 6 years and older  6 years and older  6 years and older | The recommended starting dose of Ritalin LA is 20mg once daily, when in the judgment of the clinician a lower initial dose is appropriate treatment may begin at 10mg. If required it should be increased gradually at weekly intervals up to 20 mg to 40 mg each day. The maximum dose for children is 60 mg each day.  The usual starting dose is 5 mg (half a tablet) once or twice each day. If necessary, the dose can be increased by 5 or 10 mg each week up to a maximum of 60 mg (6 tablets) each day.  The starting dose is one CONCERTA 18 mg extended-release tablet in the morning. The maximum dose is 54 mg a day taken as one dose. |
| Dexamphetamine tablets (Sigma) | Over 3 years | Treatment may be started with half a tablet (2.5mg) daily. The dose may be increased by 2.5mg every week until the required response is obtained up to a maximum of 40 mg (8 tablets) each day taken in two divided doses. |

**Stimulant medication side effects:**

- Reduced appetite —doctor may recommend giving the tablets at or after breakfast or lunch if your child is affected.
- Headaches and abdominal discomfort — these may wear off with time.
- Sleep problems.
- Dizziness, tics or raised blood pressure — these side effects tend to be rare.
